# Functional Hair Cell Mechanotransducer Channels Are Required for Aminoglycoside Ototoxicity

**DOI:** 10.1371/journal.pone.0022347

**Published:** 2011-07-26

**Authors:** Abdelrahman Alharazneh, Lauren Luk, Markus Huth, Ashkan Monfared, Peter S. Steyger, Alan G. Cheng, Anthony J. Ricci

**Affiliations:** 1 Department of Otolaryngology-Head and Neck Surgery, Stanford University, Stanford, California, United States of America; 2 Department of Special Surgery, Mu'tah University, Alkarak, Jordan; 3 Department of Otolaryngology-Head and Neck Surgery, Oregon Health & Science University, Portland, Oregon, United States of America; 4 Department of Molecular and Cellular Physiology, Stanford University, Stanford, California, United States of America; Yale University, United States of America

## Abstract

Aminoglycosides (AG) are commonly prescribed antibiotics with potent bactericidal activities. One main side effect is permanent sensorineural hearing loss, induced by selective inner ear sensory hair cell death. Much work has focused on AG's initiating cell death processes, however, fewer studies exist defining mechanisms of AG uptake by hair cells. The current study investigated two proposed mechanisms of AG transport in mammalian hair cells: mechanotransducer (MET) channels and endocytosis. To study these two mechanisms, rat cochlear explants were cultured as whole organs in gentamicin-containing media. Two-photon imaging of Texas Red conjugated gentamicin (GTTR) uptake into live hair cells was rapid and selective. Hypocalcemia, which increases the open probability of MET channels, increased AG entry into hair cells. Three blockers of MET channels (curare, quinine, and amiloride) significantly reduced GTTR uptake, whereas the endocytosis inhibitor concanavalin A did not. Dynosore quenched the fluorescence of GTTR and could not be tested. Pharmacologic blockade of MET channels with curare or quinine, but not concanavalin A or dynosore, prevented hair cell loss when challenged with gentamicin for up to 96 hours. Taken together, data indicate that the patency of MET channels mediated AG entry into hair cells and its toxicity. Results suggest that limiting permeation of AGs through MET channel or preventing their entry into endolymph are potential therapeutic targets for preventing hair cell death and hearing loss.

## Introduction

Inner ear hair cells are the mechanosensory cells essential for hearing function. As mammalian hair cells do not regenerate, damage or loss of hair cells leads to permanent hearing impairment. One common cause of hair cell death is exposure to aminoglycoside antibiotics [1], [2]. As a result, up to 25% of patients treated with aminoglycosides develop irreversible sensorineural hearing loss [3], [4]. Patients suffering from recurrent or severe infection, such as those with cystic fibrosis, are at particular high risk of such iatrogenic hearing loss [5], [6]. Entry of aminoglycosides into hair cells is necessary to induce cell death [7]. Hair cell death is thought to be mediated by reactive oxygen species [8], [9] and caspase activation [1], [2], [10], [11], although caspase-independent cell death can also occur [12]. Extensive work has characterized the intracellular events occurring after aminoglycosides enter hair cells [3], [8], [13], yet studies examining the mechanism of aminoglycoside entry into hair cells are limited.

In hair cells, aminoglycosides block mechanotransducer (MET) channels [14], [15], [16], [17], ATP receptors [18], Ca-activated K^+^ channels [19], and nicotinic acetylcholine receptors [20]. The pore size of the narrowest portion of MET channels was estimated at 1.25 nm [21], thus large enough to accommodate the passage of dihydrostreptomycin, an aminoglycoside, whose end-on diameter was estimated at 0.8 nm [22]. Electrophysiological data on mouse cochlear hair cells suggested that dihydrostreptomycin (DHS) was a permeant blocker of the MET channels *in vitro* [22]. Hypocalcemic conditions, which increase the open probability of MET channels, amplified the blocking efficacy of DHS [17] and increased neomycin's and gentamicin's toxicity in hair cells [23]. In the presence of FM1-43, a permeant blocker of the MET channels, toxicity caused by neomycin, a closely-related aminoglycoside, on mouse hair cells was reduced, suggesting the two drugs compete for entry into hair cells [24], [25].

Alternatively, AGs uptake into hair cells may be via receptor-mediated endocytosis. Endocytic pathways are present in the apical [26], [27], [28] and basolateral membranes of hair cells [29] and could provide a means of AG accumulation. The current study demonstrates that aminoglycoside entry via MET channels is primarily responsible for uptake leading to hair cell death.

## Results

### Hair cell toxicity caused by aminoglycosides

**Figure 1:**
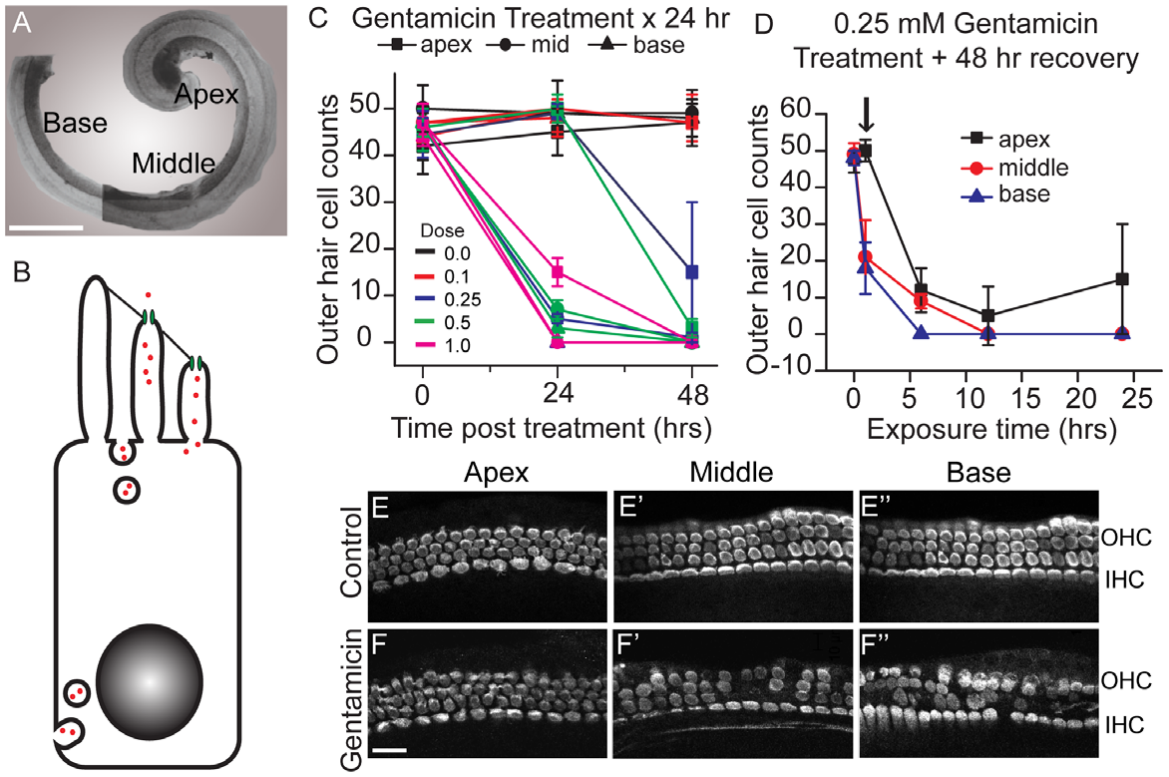
Gentamicin causes outer hair cell loss. A. Low magnification view of an isolated organ of Corti from a postnatal 4-day-old (P4) rat. B. Schematic of two proposed mechanisms of aminoglycoside (red dots) entry into hair cells via mechanotransducer channels (green) on stereociliary bundle or endocytosis (on apical or basolateral membranes). C. P4 cochleae were cultured overnight before treatment with gentamicin (0, 0.1, 0.25, 0.5, and 1.0 mM) for 24 hr, followed by 0-, 24-, or 48-hr recovery periods. Counts of phalloidin- and parvalbumin-labeled hair cells revealed that no significant hair cell loss at the end of a 24-hr treatment. With additional recovery periods, the middle and basal turns were most susceptible to hair cell loss. D. Cochleae were treated with 0.25 mM gentamicin for 1–24 hours followed by a 48-hr, antibiotic-free recovery. After 1-hr treatment, an apical-basal gradient of hair cell loss was observed. Arrow indicates the treatment paradigm chosen for the Panels E–F. Each data point represents an analysis of three to ten experiments. E–F. Representative images of control cochleae and those treated with gentamicin (0.25 mM) for 1 hr, followed by a 48-hr recovery period. Damage to parvalbumin 3-labeled hair cells was noted in the middle and basal turns of the cochlea (notably in OHCs rows 1 and 2). Error bars = S.D. Scale bars = 250 µm in A, 20 µm in C–D.

The first step in assessing AG toxicity and entry mechanisms into sensory hair cells was to develop an *in vitro* preparation where toxicity could be reproducibly assessed, keeping in mind the limited time course that was available for imaging entry. To this end, an in-depth characterization of hair cell damage as a result of gentamicin treatment was performed. Sensory hair cells along the cochlea were analyzed based on tonotopic location as apical, middle, and basal (Figure 1A). Currents of the MET channels are present in the basal turn of the cochlea at birth and this maturation process continues in culture conditions [30]. By isolating and culturing cochleae from postnatal 4-day-old (P4) pups in control media overnight, we investigated functional 5-day-old hair cells, which have MET channel conductance expressed in a tonotopic manner for outer hair cells, decreasing in a basal-apical gradient. This model system was used to directly investigate the role of mechanotransduction channels in aminoglycoside-induced hair cell death (Figure 1B). AG toxicity is dependent on exposure time, dosage and recovery time. We explored each of these in turn, first treating for 24 hrs and then allowing 0, 24 or 48 hrs of recovery for five doses of gentamicin (Figure 1C). Gentamicin was chosen because of the availability of the fluorescent conjugated compound gentamicin Texas Red (GTTR). The first experiment exposed cultures to doses from 0.1 to 1.0 mM gentamicin for 24 hrs and then allowed recovery for 0, 24 and 48 hrs (Figure 1C). The apical tissue was the least sensitive and basal cells most sensitive to treatment. Disappearance of both parvalbumin 3-labeled cell body and phalloidin-labeled stereocilia bundle of hair cells was used for quantifying hair cell loss. Given that the hair cell body can remain despite stereocilia bundle loss after gentamicin treatment [31], quantification of hair cell body number was used for the remaining experiments. Although there was little immediate cell loss following treatment by 24 hrs, basal cell loss was almost complete at 24 hr recovery while middle regions showed maximal loss for doses greater than 0.1 mM. Apical preparations showed loss of more than 60% only at the highest dose. At 48 hrs of recovery all regions and all doses showed maximal loss. This data indicates that 24 hrs of treatment is too damaging at any dose tested. We then varied the treatment time while maintaining a 48 hr recovery period. The results for the 0.25 mM gentamicin dose is presented in Figure 1D where treatment times varied from 1–24 hrs. From this data we selected the 1 hr treatment time and 48 hr recovery. This treatment gives a tonotopic loss with the base being the most severe (Figure 1D–F). Furthermore the 1 hr treatment can be used for time lapse imaging of uptake properties as well.

### Gentamicin uptake into hair cells

**Figure 2:**
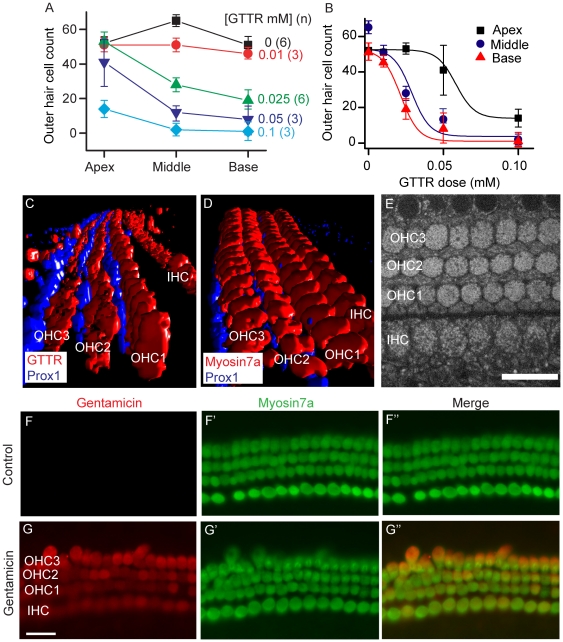
Gentamicin selectively enters hair cells. A–B. The dose-response relationship between GTTR and outer hair cell survival was determined. Cochleae were treated with GTTR for 1 hr and allowed to recover for 48 hr in drug free media. Like native (or unconjugated) gentamicin, the basal and middle turns were more susceptible to hair cells loss when treated with GTTR. C–D. GTTR (3 µM) selectively entered inner and outer hair cells and not adjacent prox1-labeled supporting cells (Deiters' and pillar cells). Staining for myosin 7a, a specific marker for outer and inner hair cells, in a P3 mouse cochlea exhibited a similar expression pattern as GTTR. E. NADH signals were detected in both outer and inner hair cells in cochlear organs deemed healthy for analysis for GTTR uptake. F–G. Cochleae cultured in the presence of gentamicin (0.1 mM for 1 hr followed by a 48 hr drug free recovery period) were immunolabeled for gentamicin, which was detected only in myosin 7a-positive hair cells. Error bars = S.D. Scale bars = 20 µm.

The Texas Red-conjugated version of gentamicin (GTTR) allows for live imaging of drug uptake in hair cells [32]. To determine the toxicity profile of GTTR in our *in vitro* preparation of rat cochlea, the dose-response relationship between outer hair cell survival and GTTR concentration was investigated. Like gentamicin, a dose-dependent outer hair cell loss was noted for GTTR, with the basal turn being the most susceptible (Figure 2A–B). Imaging for NADH signals found that both hair cells and support cells are robust prior to GTTR administration (Figure 2E). In the time frame recorded, uptake of GTTR was detected preferentially in outer and inner hair cells and not in adjacent prox1-labeled supporting cells (Figure 2C). Similar localization was observed for GTTR as for myosin 7a, again supporting selective hair cell uptake. Comparison of GTTR label with staining obtained using a specific antibody for gentamicin (Figure 2F–G) also demonstrates strong colocalization.

**Figure 3:**
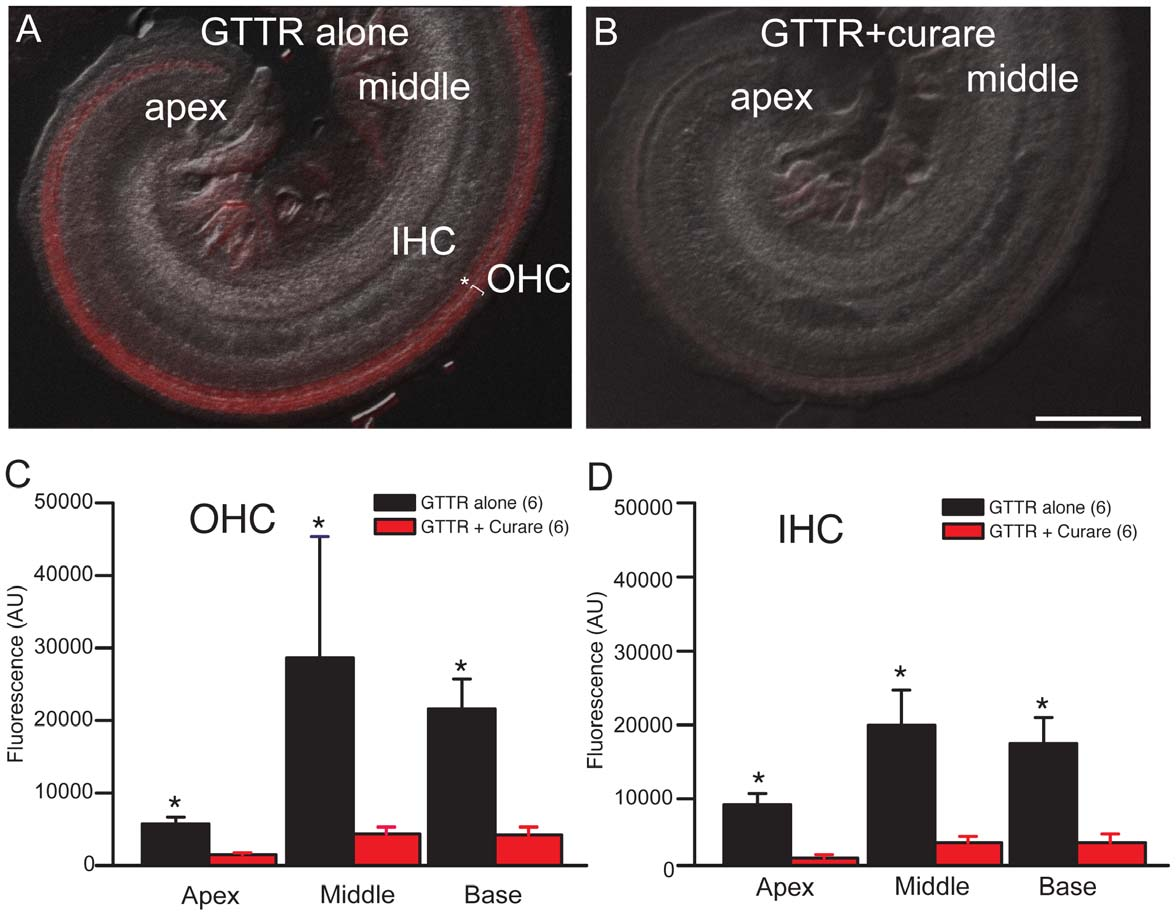
Open MET channels are required for GTTR entry into hair cells. A. Following incubation in control media for 24 hr, P3 rat cochleae treated with GTTR alone (3 µM for 1 hr at 37°C) showed robust uptake in inner (asterisk) and outer hair cells (OHC) in the middle and basal cochlear turns. B. Co-treatment with curare (1 mM) effectively prevented GTTR uptake. C–D. Quantification of GTTR fluorescent intensity demonstrated that uptake is significantly reduced by blocking MET channels with curare in both the inner and outer hair cells throughout the cochlear turns. Error bars = S.D. Scale bars = 100 µm.

To determine if entry through MET channels was a significant pathway, organ of Corti cultures were incubated at 37°C with GTTR for 1 hr in the absence and presence of the open channel blocker curare (Figure 3). Figure 3A shows the apical and middle turns where IHC and OHCs were selectively labelled. Some labelling also appears in the remaining portions of Reissner's membrane. Curare, significantly reduces the labelling in hair cells. Figure 3C–D summarizes the uptake as measured in OHCs (C) and IHCs (D). Curare significantly reduced uptake in both cell types. We next further investigated the time course of GTTR uptake under two-photon microscopy.

**Figure 4:**
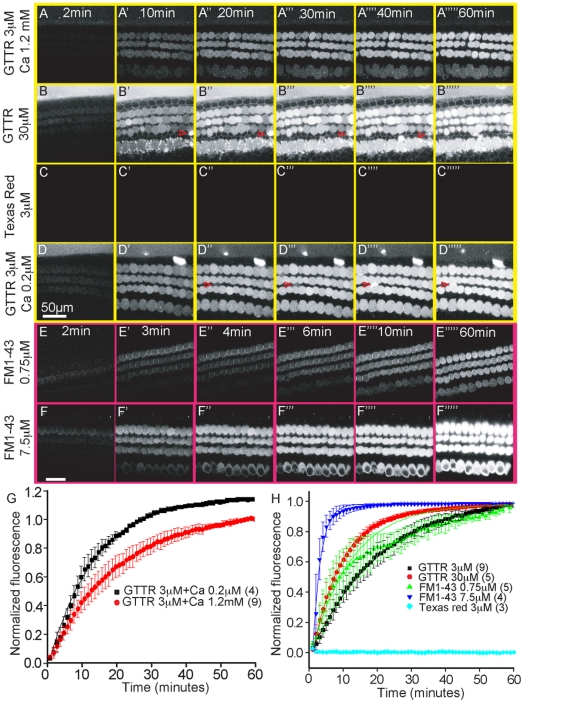
Two-photon imaging for GTTR uptake in live cochlear hair cells. P3 cochleae were cultured for 24 hr before being treated with GTTR or FM1-43 for an hour at room temperature. A–H. GTTR (3 µM) rapidly entered hair cells, whereas Texas Red alone (3 µM) did not. Both high dose GTTR (30 µM) and hypocalcemic conditions promoted GTTR entry into hair cells. In these 2 conditions GTTR was rarely observed outside hair cells in a delayed (>10 min) fashion (arrowheads). E–F. The amphiphatic dye FM1-43 has been shown to be a permeant blocker of the MET channel. The entry of FM1-43 (0.75 µM) (E–F in pink background) into hair cells was more rapid (note different time scale) than GTTR (A–D in yellow background) and was also promoted by increasing its concentration (7.5 µM). Error bars = S.D. Scale bar = 20 µm.

Two-photon imaging was used to monitor uptake of GTTR into cultured organ of Corti. The thin focal planes obtained with two-photon microscopy allowed for imaging in the presence of GTTR-containing media, while also limiting photobleaching and phototoxicity. Prior to GTTR application, endogenous fluorescent emission of NADH was observed as an indication of tissue viability (Figure 2E). A lack of NADH signal was interpreted as poor tissue viability and those organs were discarded prior to experimentation. At a normal extracellular calcium concentration (1.2 mM), GTTR was rapidly taken up into the outer hair cells (t = 21.0±0.2 min; Figure 4A). A time lapse video of uptake can be found in Movie S1. This movie illustrates the selectivity of GTTR for hair cells. When applied alone, Texas Red was not detected in either hair cells or supporting cells in the organ of Corti (Figures 4C and H). GTTR (3 µM) demonstrated a steep rate of uptake in the first 30 minutes (Figures 4G–H). Hypocalcemic conditions, which increased the open probability of MET channels [33], [34], raised the rate of GTTR uptake (t = 13.5±0.2 min; Figures 4D and G). Increasing the concentration of GTTR (to 30 µM) also sharply elevated its rate of uptake into hair cells (t = 9.0±0.5 min; Figures 4B and H). No significant GTTR uptake was detected in surrounding supporting cells when GTTR (3 µM) was applied in media containing 1.2 mM [Ca^2+^ ] (Figure 4A). Lowering extracellular [Ca^2+^ ] or raising GTTR concentration did not increase GTTR uptake into supporting cells, suggesting that they are not equipped with the mechanisms needed for rapid GTTR uptake present in inner and outer hair cells. GTTR was observed outside hair cells in a delayed fashion (>10 min) both under hypocalcemia (arrowheads in Figure 4B) and high dose GTTR (30 µM) treatment (arrowheads in Figure 4D). This observation is possibly related to leakage of GTTR into the intercellular space as result of damage to the hair cells. Otherwise, the lack of GTTR between cells (particularly OHCs) suggests tight compartmentalization and limited diffusion. Punctate labelling near the basolateral aspects of inner hair cells was occasionally observed when GTTR was administered and was not affected by co-treatment with blockers of MET channels or endocytosis. One possible explanation for these punctate labelling is direct uptake into nerve terminals immediately adjacent to inner hair cells, as gentamicin has been detected in spiral ganglia [35]. If true, the mechanism for this uptake remains to be explored.

In many cells, the amphipathic styryl dye FM1-43 is internalized via endocytosis [36]. However, its rapid entry into hair cells depends upon open MET channels [24], [25], thus making it a useful compound to compare kinetics with GTTR. When applied at 0.75 µM, its entry into hair cells was rapidly detected in outer hair cells (t = 12.0±0.5 min; Figures 4E and H). Its rate of entry steeply increased when the concentration was increased to 7.5 µM (t = 2.4±0.2 min; Figures 4F and H). The difference in entry rates between FM1-43 and GTTR is likely related to the higher affinity of FM1-43 for the MET channel [17], [25].

**Figure 5:**
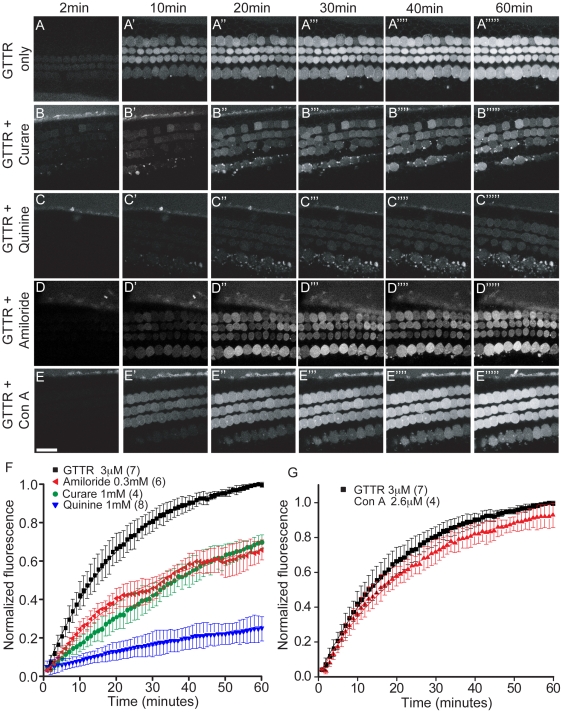
Kinetics of GTTR uptake with blockers of MET channel or endocytosis. A–G. Both curare (1 mM) and amiloride (0.3 mM) reduced GTTR uptake into outer hair cells, but an initial rise in GTTR uptake, like that seen when cochleae were treated with GTTR alone, was still observed despite amiloride co-treatment. Quinine (1 mM) was the most effective in limiting GTTR uptake among all the drugs tested. No significant change in GTTR uptake was seen with concanavalin A (2.6 µM) treatment. Error bars = S.D. Scale bar = 20 µm.

To assess the effects of MET channel conductance and endocytosis on the rate of GTTR uptake into hair cells, MET channel blockers representing different chemical classes were used: curare, quinine, and amiloride. GTTR alone entered outer hair cells at a rapid rate (t = 24±1 min), plateauing during the 60 minutes of treatment. For the following experiments, the intensity of fluorescence at 60 minutes (F_60_) was normalized to this value. Co-treatment with MET channel blockers effectively slowed and reduced uptake of GTTR into hair cells (Figures 5B–C, F). At the concentrations tested, quinine (t = 246.0±4.0 min, 24±7 normalized F_60_) and curare (t = 83.0±5.0 min, 70±4 normalized F_60_) were both effective in blocking GTTR uptake. When amiloride was simultaneously applied, an initial phase of GTTR uptake as rapid as that of GTTR alone was observed, followed by a second phase of slower uptake (t_1_ = 21±1.0 min and t_2_ = 135.0±7.0 min, 65±5 normalized F_60_). That GTTR uptake was not completely antagonized is a function of the competitive nature of the block and does not provide evidence for an alternate uptake mechanism.

To more directly test endocytosis as an alternative pathway, concanavalin A and dynosore were tested as nonselective blockers of endocytosis. The rate and final amount of GTTR uptake in outer hair cells were not significantly altered by the co-application of concanavalin A (Figure 5; t = 24±1.0 min; 92±7 normalized F_60_). The effect of dynosore treatment on GTTR uptake could not be determined because it effectively quenched the fluorescence of GTTR upon application (see Figure S1). Taken together, GTTR entry into hair cells was highly dependent on the patency of MET channels.

**Figure 6:**
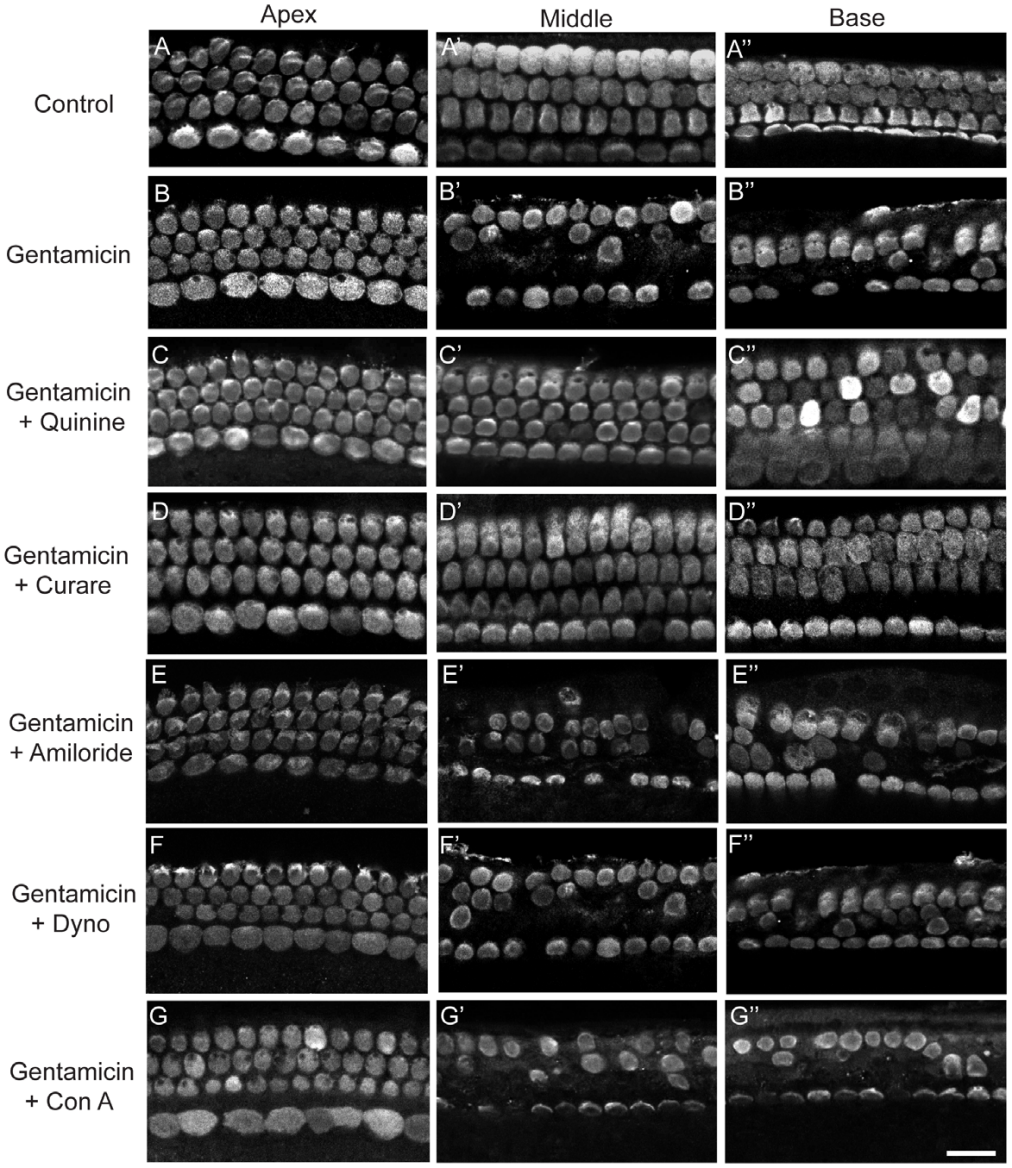
Blockers of MET channels, but not endocytosis, protect hair cells from gentamicin toxicity. A–G. Cochleae were harvested from P4 rats and cultured in antibiotic-free media for 24 hr before treated with gentamicin (0.1 mM) for 1 hr, followed by a 48-hr antibiotic-free recovery period. Tissues were fixed and labeled with parvalbumin 3 antibody. Representative confocal images from each region of the cochlea are shown. When applied concurrently with gentamicin, both curare (1 mM) and quinine (0.5 mM), improved hair cell survival. No significant protection was observed with a different MET channel blocker, amiloride (300 µM), or when blockers of endocytosis (concanavalin A 2.6 µm or dynosore 80 µM) were co-administered. Scale bar = 20 µm.

**Figure 7:**
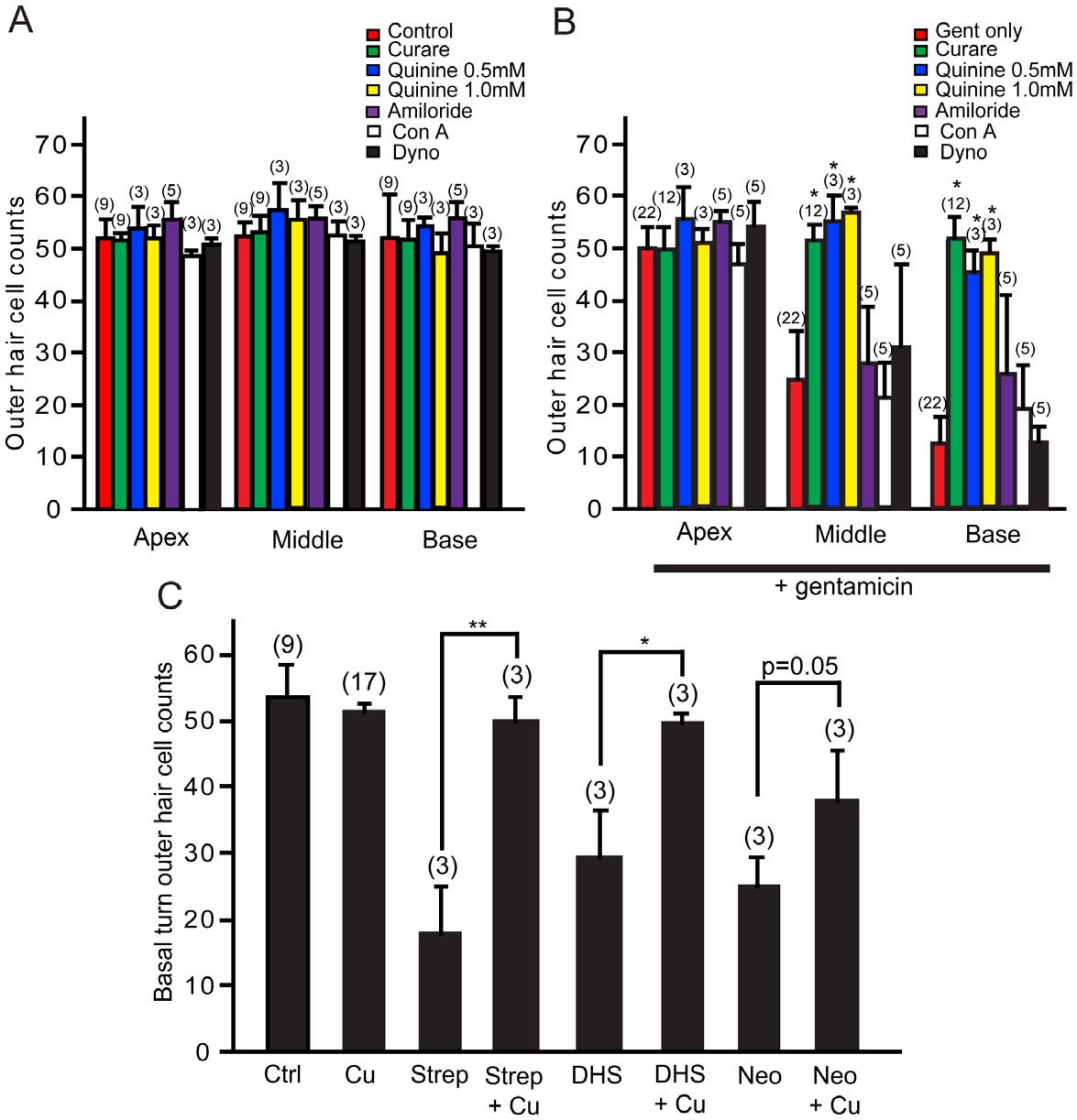
Quantitative analysis of outer hair cell survival. A. Curare, quinine, amiloride, concanavalin A, and dynosore were administered individually to rat cochlear cultures and found not to affect outer hair cell survival (determined by parvalbumin 3 labeling). B. Both curare and quinine provided significant protection to outer hair cells from gentamicin damage in both the basal and middle turns of the cochlea. No significant protection from gentamicin toxicity was observed when cochleae were co-treated with amiloride, concanavalin A or dynosore. C. Curare significantly improved outer hair cell survival in the basal turn of cochleae exposed to various aminoglycosides, including streptomycin and dihydrostreptomycin, but not neomycin. Error bars = S.D. * = p<0.001, ** = p<0.01.

To determine the role of mechanotransduction in aminoglycoside-induced hair cell toxicity, the same MET channel blockers were used to limit aminoglycoside toxicity in the following experiments. Cochlear cultures were co-treated with gentamicin and the following MET blockers: quinine, curare, AM1-43, and amiloride. The experimental design was to treat with aminoglycoside alone, antagonist alone, or to co-treat with both compounds. Only experiments where the dose of antagonist did not damage cochlear cultures were included. Administration of AM1-43 (0.25–1.0 µM) proved to be toxic to the sensory epithelium and was therefore excluded from further analysis. When co-administered with gentamicin, both quinine and curare conferred protection against hair cell loss (Figure 6, Figure 7). Hair cell survival was significantly improved by quinine doses of 0.5 mM (92.6% vs. 23.5% in the basal turn; p<0.001) and 1.0 mM (99.6%; p<0.001) and by curare 1.0 mM (99.6%; p<0.001; Figure 6, Figure 7). Although a trend towards improved hair cell survival was observed with co-treatment with amiloride, the degree of protection was variable and did not reach statistical significance (60%; p = 0.4; Figure 6, Figure 7).

When curare was simultaneously applied with other aminoglycosides, significantly improved hair cell survival was observed (see Figure 7C). For groups treated with streptomycin (hair cell survival improved to 98% from 35%; p<0.01) and dihydrostreptomycin (hair cell survival improved to 102% from 42%; p<0.001). Only a trend towards improved hair cell survival was noted in the neomycin-treated group (hair cell survival: 78% as compared to 55%; p = 0.05). These data suggest that aminoglycosides are similarly entering hair cells via MET channels, the different efficacy likely relates to how well curare competes with the AG or differences in toxicities of the compounds after entry into hair cells. By constructing molecular models of the tested aminoglycosides (streptomycin, dihydrostreptomycin, neomycin, and gentamicin), end-on diameters were estimated to be smaller than the proposed MET channel pore size of 1.25±0.08 nm [21]: 1.04 nm (streptomycin and DHS), 0.98 nm (gentamicin), and 0.96 nm (neomycin).

To determine whether endocytosis played a similar role in aminoglycoside uptake, several inhibitors of this process were also tested. When concanavalin A (Con A; 2.6 µM), a general inhibitor of endocytosis, was co-administered with gentamicin, no significant protection of hair cells was observed (basal turn: 36%; p = 0.2; Figure 6, Figure 7). Inhibition of dynamin by dynosore (80 µM) prevents clathrin-mediated endocytosis [37]. Dynosore did not improve hair cell survival when challenged by gentamicin treatment (basal turn: 24% survival; p = 0.3; Figure 6, Figure 7). Phenylarsine oxide another inhibitor of endocytosis proved to be toxic and so could not be evaluated. Taken together, blockers of MET channels provided significant protection of gentamicin-induced outer hair cell loss, whereas inhibition of endocytosis did not.

**Figure 8:**
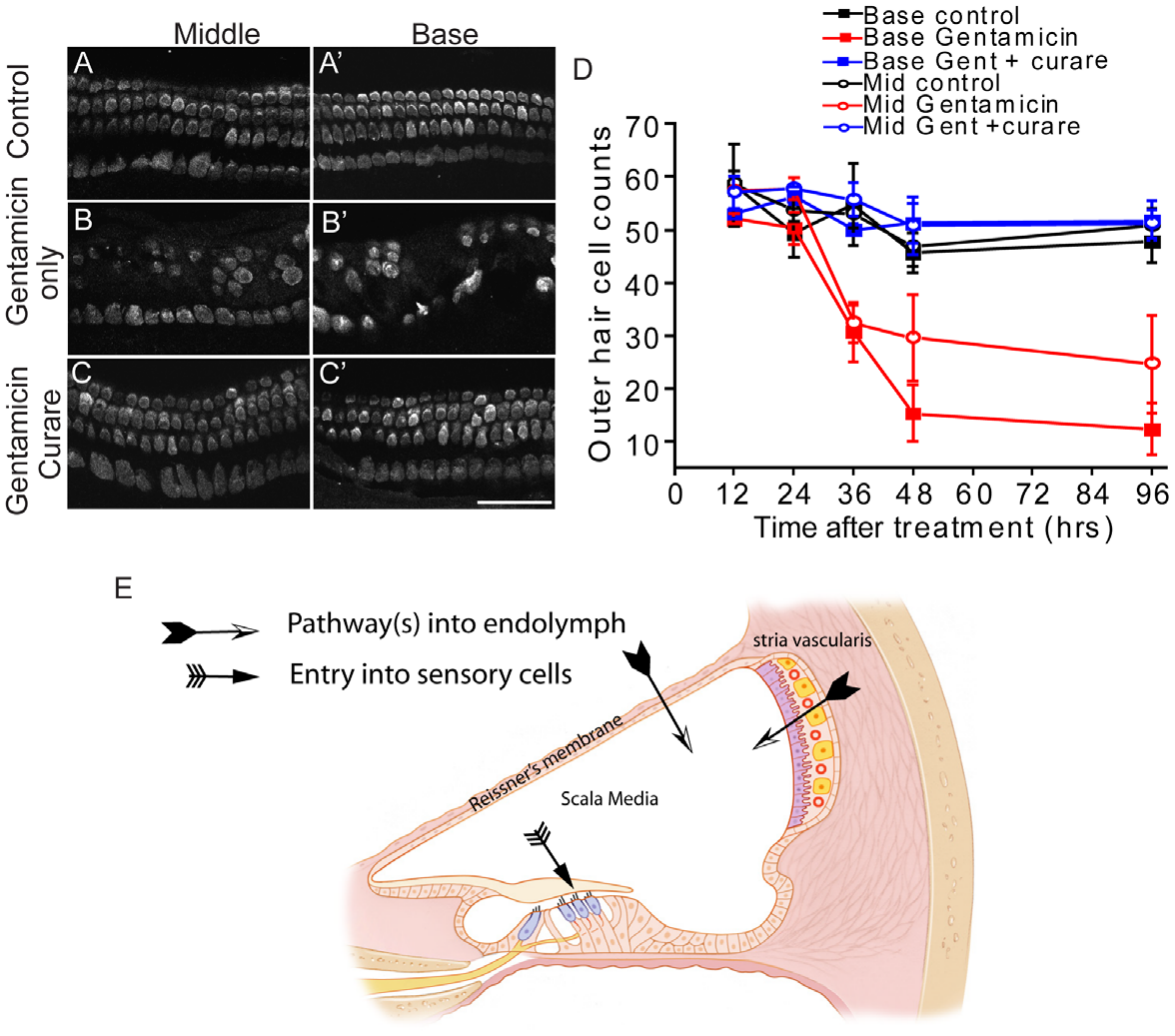
Curare protected hair cells in long-term cultures. A–C. Cochleae treated with gentamicin (0.1 mM) with or without curare (1.0 mM) remained in gentamicin-free media for four additional days. Survival of outer hair cells (determined by parvalbumin 3 labeling) in the curare-treated cochleae was comparable to that in untreated, cultured cochleae. D. Quantitative analysis of the gentamicin-treated cochleae demonstrated a slight decline in outer hair cell survival 96 hr versus 48 hr after treatment. Cochleae co-treated with curare and gentamicin have comparable numbers of outer hair cells at both time points. Each data point represents an analysis of three to twenty-two experiments. E. Schematic of possible entry pathways for aminoglycoside first into the scala media from the stria vasculars or Reissner's membrane, then into sensory hair cells in the inner ear. Error bars = S.D. Scale bar = 50 µm.

To test whether MET channel blockers prevented or delayed hair cell death, cultures were maintained for up to 96 hours post treatment. Figure 8 illustrates these results, demonstrating that hair cell survival was enhanced even after an extended recovery period (100% vs. 32%; p<0.001). It should be noted that changing the experimental paradigm could alter the efficacy of the channel blockers. Given that the aminoglycosides are competing with the channel blockers and the channel blockers are not irreversibly binding to the channel, longer exposure times or higher doses of aminoglycosides would be expected to overwhelm the blocking agents. Competitive blocking of the channel is expected to slow, not prevent entry; therefore, at equilibrium a similar amount of aminoglycoside is expected to have entered the cell, what is different is the time it takes to reach equilibrium. However, this was not observed, as MET channel blockers did effectively prevent hair cell death.

## Discussion

A serious side-effect of aminoglycoside antibiotics is irreversible sensorineural hearing loss, which occurs in up to 1 in 4 treated patients [3]. By entering and damaging cochlear hair cells, aminoglycosides cause hair cell death and consequently hearing loss. One possible intervention is to reduce or prevent aminoglycoside uptake into sensory hair cells. The current study was designed to investigate the effects of perturbing two proposed mechanisms of aminoglycoside uptake on hair cell survival.

Similar to previous results using *in vitro* and *in vivo* models [38], [39], we found basal outer hair cells to be the most susceptible to damage from gentamicin *in vitro*. This susceptibility may be explained in part by the higher open probability and single channel conductance of MET channels in hair cells residing in this region [40]. But lower levels of antioxidants among basal hair cells have also been proposed to be responsible for this differential susceptibility [38]. At the early postnatal ages tested, the open probability of the MET channel is the highest in the basal turn, and decreases in a basal-to-apical gradient [30]. All the aminoglycoside antibiotics tested, including GTTR, resulted in a similar damage profile, suggesting a common mechanism of damage and entry into hair cells among these molecules. In support of this notion, we observed loss of apical hair cells with prolonged (24 hr) or high concentration (1.0 mM) of gentamicin treatment, suggesting that increasing the driving force of gentamicin entry into hair cells can overcome a lower open probability of MET channels in apical hair cells [30]. Our observation is in agreement with other damage protocols that yielded nearly complete outer hair cell loss [39], [41].

GTTR uptake into hair cells was enhanced by hypocalcemic conditions, which increases the open probability of the MET channel [34], [42]. This observation is in agreement with the previous findings that hypocalcemia increased the toxicity of aminoglycosides to hair cells [23]. Similarly, treatment paradigms that decrease the open probability of the MET channel, such as hypercalcemia and myosin 7a deficiency, conferred protection to hair cells from aminoglycosides [23], [28]. Alternatively, quinine and curare provided hair cells with protection by blocking the MET channels and thus preventing aminoglycoside entry. Our results are consistent with previous findings that compounds that block aminoglycoside uptake into hair cells are protective [43]. Unlike curare and quinine, amiloride was not as potent at protecting hair cells from gentamicin toxicity. Although it decreased GTTR uptake into hair cells, an initial phase of rapid uptake, comparable to gentamicin alone was observed followed by a slowing of entry, leaving a final GTTR fluorescence comparable to that of curare co-treatment. It is possible that amiloride, which actually binds to the channel and is not an open or permeable blocker, was less effective in limiting the rate of aminoglycoside entry, which may be an important factor for its ultimate toxicity [44]. In light of the extensive body of work demonstrating that aminoglycosides cause a rapid increase in reactive oxygen species in hair cells [8], the rate of aminoglycoside entry into hair cells may be important as the levels of antioxidants in hair cells are limited and may be overwhelmed analogous to any other buffering system [38].

Our results suggest that endocytosis does not play a major role in gentamicin–induce hair cell toxicity. The pharmacology for blocking endocytosis is limited and not very specific given the multiple endocytic mechanisms. It is possible that endocytosis mediates some uptake of aminoglycosides, which can be detected in subcellar compartments such as lysosomes and endoplasmic reticulum [27], [45], [46]. It is also possible that compartmentalization occurs after entry. Another limitation of our current study is that we cannot rule out the possibility that other transport mechanisms might also contribute to AG uptake under other pathologic conditions, such as those by other channels [47]. It would be interesting to elucidate these modes of entry and role of compartmentalized aminoglycosides in future studies.

Although the role of endocytosis may be underestimated, the importance of MET channels is clear. Given the striking protection observed under conditions where the MET channels were largely biased closed, the role of the MET channels may be even more important than presented. Under physiological conditions where calcium is low in the endolymph, the probability of opening of MET channels is much larger than tested here. Also entry of the AGs is driven electrically. Present studies simply used existing membrane potential of the hair cells in culture, rather than increasing driving forces presented by the endocochlear potential and the endolymph solution, or in the case of the GTTR experiments, the warmer temperature. Under more physiological conditions the MET channel open probability is predicted to be closer to 0.3–0.5 as compared to culture condition where it is 0.05–0.1 [42] and the current amplitudes *in vivo* would be predicted to increase close to an order of magnitude, thus uptake via this mechanism is predicted to be greatly enhanced. The one caveat to this argument is that AGs need to enter the endolymph compartment in order to access the MET channels.

Previous studies have demonstrated that the level of aminoglycoside in the inner ear endolymph does not reach that of plasma, but its clearance time is extremely slow (>30 days) [48]. As steady state levels are a function of both entry and clearance, it is possible that lower endolymph levels reflect high rates of entry into hair cells reducing steady-state values. Alternatively the lower levels in endolymph may explain the lack of immediate hearing loss due to blockage of MET channels prior to hair cell damage. Given that hair cells do not metabolize the AG, accumulation will be cumulative so low levels in endolymph do not reduce the likelihood of entry via MET channels. Studies that examine the kinetics of aminoglycoside entry into the inner ear and sensory hair cells *in vivo* will help shed light on these possible mechanisms.

Although the molecular composition of the MET channel remains elusive, its pharmacologic and electrophysiologic properties have been characterized [21], [22], [33], [34], [49]. Within the inner ear, only the sensory hair cells exhibit properties of the MET channels. The narrowest portion of the MET channel was estimated at 1.25±0.08 nm [21], thus large enough to accommodate the passage of the aminoglycoside dihydrostreptomycin [22] and the styryl dye FM1-43 [24], [25]. Measurements of the end-on diameters of streptomycin, dihydrostreptomycin, neomycin and gentamicin also found them small enough to pass through these channels. When applied to cochlear hair cells *in vitro*, both gentamicin and GTTR (Figure 2) are selectively taken up by outer and inner hair cells, but not in adjacent supporting cells. The end-on diameter of GTTR was estimated to be 1.47 nm and thus larger than the reported MET channel pore size (1.25 nm) [21]. Two possible explanations for the observed GTTR uptake into hair cells are first that the MET channel pore size has been underestimated, and second that the MET channel pore temporarily dilates during GTTR entry. In the latter scenario, such a dynamic and reversible dilation has been characterized in other nonselective cation channels [50], [51]. The proposed characteristics are hypothetical and warrant further investigation.

Uptake of gentamicin via receptor-mediated endocytosis was found to occur in proximal renal tubular cells requiring the receptor megalin [52]. While megalin expression is found in the stria vascularis and is notably absent in sensory hair cells [53], whether it similarly binds and transports aminoglycosides in the cochlea is not yet determined. This mechanism of transport in the stria vascularis would be particularly interesting since this is the site where endolymph is produced and aminoglycoside are thought to enter the labyrinth [32]. Inhibiting entry into endolymph may prevent entry into hair cells via MET channels (Figure 8E). Given that MET channels are located atop the stereocilia hair bundles bathed in the scala media, an enclosed fluid compartment with no direct access to a blood supply, aminoglycosides must be transported into endolymph, likely from the stria vascularis [32]. Preventing aminoglycoside entry into the endolymph compartment therefore would be expected to limit toxicity. Alternatively, modifying aminoglycosides to sterically limit their entry into MET channels should also be expected to alleviate ototoxicity. More studies are necessary to validate this potential benefit, including experiments in the whole animal.

In conclusion, hair cell susceptibility to aminoglycoside antibiotics stems from the rapid accumulation of these compounds into the sensory cells because of the novel access via mechanotransducer channels. Preventing access to these channels or permeation of these channels may be viable therapeutic approaches to improving the value of aminoglycosides.

## Materials and Methods

### Animals and cochlear cultures

Sprague-Dawley rats and C57BL/6 mice were obtained from Charles River laboratory (Cambridge, MA) and Jackson Laboratory (Bar Harbor, ME). All procedures involving animals were approved by the Stanford University committee on animal research (Assurance number A3213-01, Protocol ID 18606).

### Tissue cultures

Cochleae were freshly isolated from rat pups. The temporal bones with stria vascularis and modiolus were removed. Whole mount cochleae were then placed onto 10 mm coverslips (Fisher Scientific, Pittsburgh, PA) pre-coated with CellTaK (BD Biosciences, San Jose, CA), and incubated in Dulbecco's Modified Eagle Medium/F12 (Invitrogen, Carlsbad, CA) supplemented with 10% FBS and ampicillin (50 µg/ml; Sigma, St. Louis, MO) for 1–5 days at 37°C in a 5% CO_2_ atmosphere. Culture media was replenished every 1–2 days. The following drugs were tested: gentamicin, streptomycin, dihydrostreptomycin, neomycin, quinine, amiloride, concanavalin A, dynasore, phenylarsine oxide (all from Sigma), curare (Fisher Scientific), AM1-43 and FM1-43 (Invitrogen). Functional postnatal-day-5 cochleae were specifically chosen for our experiments because most apical and basal hair cells carry transduction currents at this age [30].

### Immunohistochemistry and image analyses

Cochlear organs were fixed in 4% PFA for 30 minutes at room temperature (RT), and then immersed in blocking solution [5% goat or donkey serum, 0.1% triton X-100, 1% bovine serum albumin (BSA), and 0.02% sodium azide (NaN_3_) solution in phosphate buffered solution (PBS) for 1 hour at RT. They were then incubated with primary antibodies in blocking solution overnight at 4°C in a humidified chamber. The following day, tissues were rinsed with PBS three times, and then incubated with secondary antibodies (0.1% triton X-100, 1% BSA, and 0.02% NaN_3_ solution in PBS) for 1 hour at RT. For labelling for filamentous actin, fluorescently-conjugated phalloidin (Sigma) was applied in 0.1% triton X-100 in PBS for 1 hour. After washing with PBS, tissues were mounted in antifade Fluorescence Mounting Medium (DAKO, Carpinteria, CA) and coverslipped. The following antibodies were used: anti-myosin 7a (1∶1000; Proteus Bioscience, Ramona, CA) [39]; anti-Prox1 (1∶1000; Millipore, Billerica, MA) [54]; anti-parvalbumin 3 (1∶1000) [55]; anti-gentamicin (1∶200; QED Bioscience, San Diego, CA) [56]. The specificity of these antibodies has been previously determined in the referenced studies and/or by their respective suppliers. The respective secondary antibodies were conjugated to either FITC, TRITC or Cy5 (1∶200; Jackson ImmunoResearch, West Grove, PA). Images were acquired using epifluorescent or confocal microscopy (Axioplan 2, Zeiss, Germany) and analyzed with Photoshop CS4 (Adobe Systems, San Jose, CA). The cochlea was divided into apical, middle and basal turns, and separately analyzed. At least 2 representative areas were sampled from each turn for cell quantification. Student t-test was used for statistical comparison. Three-dimensional reconstruction of z-stack images was processed using the Volocity software (v5.3.0; Improvision, Waltham, MA).

### Two-photon live imaging

Cochlear organs were cultured overnight as described above, then washed with L-15 (Sigma) before imaging. Gentamicin sulfate (Sigma) and succinimidyl esters of Texas Red dye (Invitrogen) were conjugated to compose GTTR, which was purified before use [32]. GTTR (3–30 µM) and FM1-43 (0.75–7.5 µM) were directly added to cochlear cultures. In experiments testing blockers of MET channels or endocytosis, compounds were concurrently added with GTTR. An Olympus BX-61 (Olympus, Center Valley, PA) coupled to the Prairie Ultima two photon laser scanning microscope with a 100×, 0.9 numerical aperture water immersion objective (Olympus LUMPlan) was used to image the middle turn of the organ of Corti. A 520 nm long-pass dichroic filter (Chroma Technology, Bellows Falls, VT) was used to separate the fluorescence emission into two channels and detected by photomultiplier tubes. Using Prairieview software at 1.4× magnification (Prairie Technologies, Middleton, WI), 60-minute time series were collected at 1-minute intervals. Femtosecond pulses of 900 nm illumination from a tunable Chameleon XR laser (Coherent, Santa Clara, CA) were scanned across the sample using galvanometers, power was regulated with a pockel cell. Red fluorescence originating from GTTR was isolated using a bandpass filter (620/60, Chroma Technology). Prior to adding GTTR, samples were scanned at 740 nm and blue NADH intrinsic fluorescence detected with a bandpass filter (480/30) to confirm the viability of the sample [57] (see Figure 2E). Samples with low NADH signals were discarded.

### Data Analysis

To quantify the fluorescence intensity, at least ten individual cells were selected as regions of interest (ROIs) in each T-series. Using Image J software (NIH), the pixels in the ROIs were averaged to determine the total fluorescence intensity. The average background intensity was negligible and therefore was not subtracted from the total fluorescence. The fluorescence for the ten cells was averaged to create a single average fluorescence per T-series. The average of each T-series was normalized to the maximum fluorescence (from experiments using 3 µM GTTR in 1.2 mM calcium) and this normalized average and the standard deviation was plotted as a function of time (OriginLab, Northampton, MA) to determine the average increase in cellular fluorescence over time after GTTR administration (see Figure 4G). In experiments measuring FM1-43 uptake, the average of each T-series was normalized to the maximum fluorescence from experiments using FM1-43 at 0.75 µM (see Figure 4H). Best fit curves were produced using the formula Y = 1−Ae^(−*x*/^_t_^)^, from which the time constants were calculated. Y = 1.2−Ae^(−*x*/^_t_^)^ was used for experiments examining hypocalcemic conditions and Y = 1−(Ae^(−*x*/^_t1_^)^+Be^(*x*/^_t2_^)^ for experiments examining amiloride.

### Chemical models

Drugs were reconstructed using CPK precision Molecular Models (Ealing, South Natick, MA).

## Supporting Information

Figure S1 **Dynosore quenches fluorescence of GTTR.** A. Co-treatment with GTTR (3 µM) and dynosore (80 µM) effectively made GTTR undetectable. B. When GTTR was first added and allowed to enter hair cells and dynosore was later added (15 min), an abrupt plateau of GTTR fluorescence was observed. Error bars = S.D.
(TIF)

Movie S1 **Time lapse movie of GTTR uptake.** Cochleae harvested from P4 rats were cultured overnight before being treated with GTTR (3 µM). Images were captured every minute for a total of 1 hr under two-photon microscopy.
(MOV)
