# Supplementary figures and images for: Functional Hair Cell Mechanotransducer Channels Are Required for Aminoglycoside Ototoxicity
Source: PLoS One. 2011 Jul 26;6(7):e22347. doi: 10.1371/journal.pone.0022347 (PMC3144223; doi:10.1371/journal.pone.0022347)

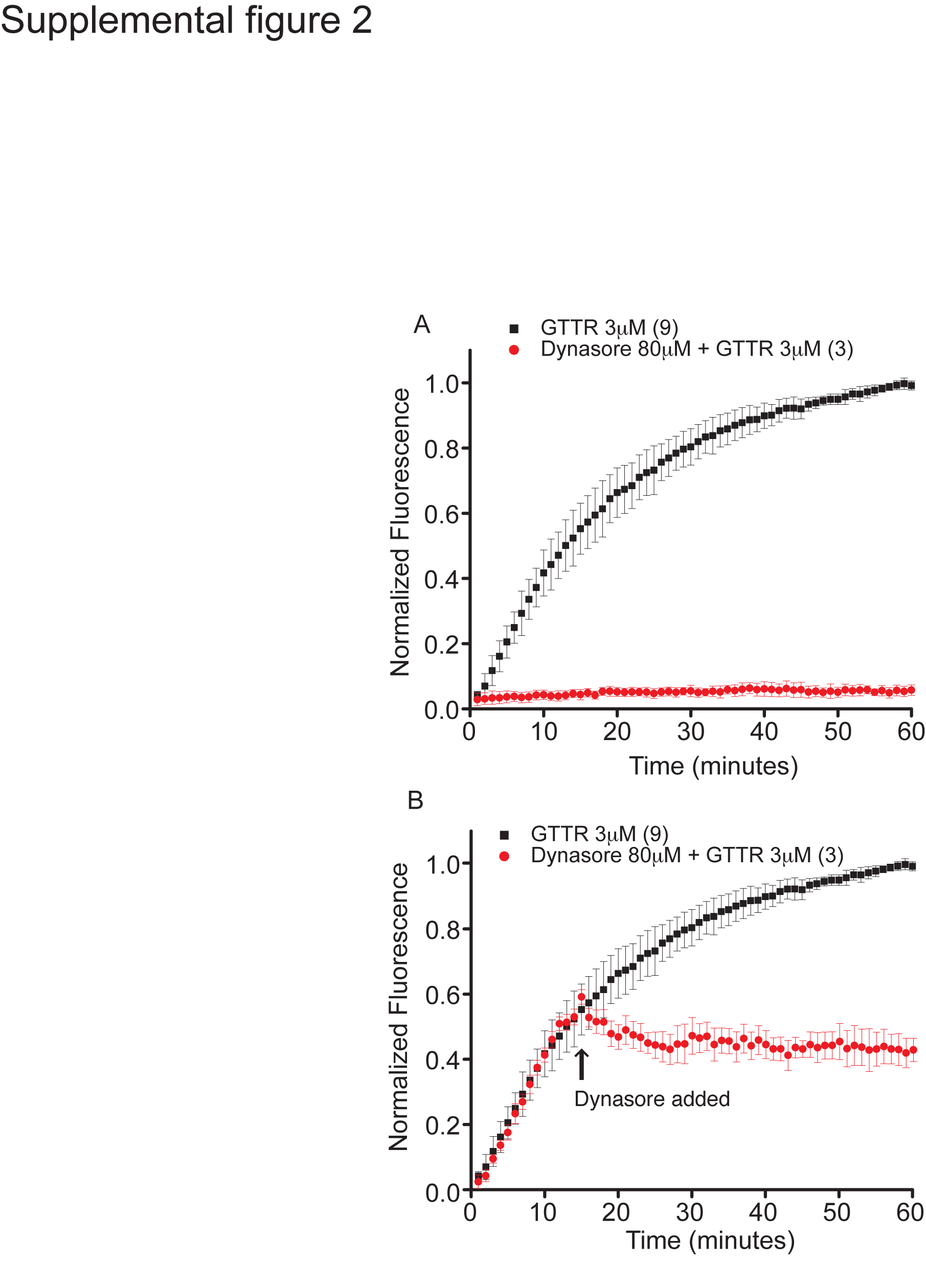

Supplement: Figure S1 — Dynosore quenches fluorescence of GTTR. A. Co-treatment with GTTR (3 µM) and dynosore (80 µM) effectively made GTTR undetectable. B. When GTTR was first added and allowed to enter hair cells and dynosore was later added (15 min), an abrupt plateau of GTTR fluorescence was observed. Error bars = S.D. (TIF) [file pone.0022347.s001.tif]
